# The Effects of Sa-Am Acupuncture Treatment on Respiratory Physiology Parameters in Amyotrophic Lateral Sclerosis Patients: A Pilot Study

**DOI:** 10.1155/2013/506317

**Published:** 2013-09-11

**Authors:** Sangmi Lee, Sungchul Kim

**Affiliations:** ^1^Department of Acupuncture & Moxibustion, Wonkwang University Korean Medical Hospital, 543-8 Juwol Dong, Nam-gu, Gwangju 503-310, Republic of Korea; ^2^ALS Center of Wonkwang University Korean Medical Hospital, 543-8 Juwol Dong, Nam-gu, Gwangju 503-310, Republic of Korea

## Abstract

Respiratory dysfunction and complications are the most common causes of death in amyotrophic lateral sclerosis. This is a pilot study to observe the changes in respiratory physiology parameters after Sa-am acupuncture treatment. Eighteen ALS patients received Sa-am acupuncture treatment twice a day for 5 days. The EtCO_2_, SpO_2_, RR, and pulse rate were measured for 15 min before and during treatment, using capnography and oximetry. Correlation of K-ALSFRS-R scores against measured parameters showed that patients who had high K-ALSFRS-R scores had greater changes in pulse rate after acupuncture stimulation; they also showed a decrease in EtCO_2_, RR, and pulse rate and an increase in SpO_2_. A comparison of the mean values of these different parameters before and after Sa-am acupuncture stimulation revealed statistically significant differences (*P* < 0.05) in SpO_2_ and pulse rate, but none in EtCO_2_ and RR. Sa-am acupuncture treatment on ALS patients seems to be more effective in the early stages of the disease. In light of increased SpO_2_ values, Sa-am acupuncture appears to have a greater effect on inspiration rather than on expiration. As a pilot study of acupuncture on ALS patients, this study could be used as a basis for future research.

## 1. Introduction

Amyotrophic lateral sclerosis (ALS) is a common progressive, neurodegenerative disorder of the voluntary motor system that affects motor neurons in the cerebral cortex, brain stem, and spinal cord [1]. It is characterized by progressive neuromuscular atrophy with early involvement of the respiratory system. The latter rapidly leads to pulmonary compromise requiring mechanical ventilation and represents the major cause of mortality [2]. Eighty-four percent of ALS patients die of respiratory complications and respiratory insufficiency in 2-3 years after diagnosis. On a retrospective chart review, it was found that 2.7% of patients with ALS had respiratory symptoms as their first symptom and that the average survival period of these patients was only 2 months [3, 4]. Hence, reduced ventilation results, in part, from progressive motor neuron degeneration, which leads to respiratory muscle weakness. Respiratory muscle weakness is defined as the inability of the respiratory muscles to generate normal levels of pressure and air flow during inspiration and expiration [5]. This leads to respiratory insufficiency, which is defined as inadequate pulmonary ventilation to the point that gas exchange is impaired, resulting in carbon dioxide retention, hypoxemia, and respiratory failure [5, 6]. ALS patients are able to withstand respiratory muscle weakness by using other muscles at the initial stage, but the symptoms progress gradually to respiratory insufficiency and eventual respiratory failure. In the majority of cases, death is related to respiratory events. The time of progression and the degree of respiratory muscle weakness are, therefore, important prognostic factors for ALS patients. Hypoventilation in ALS patients is due to respiratory muscle weakness and is associated with poor survival, cognitive impairment, and a poor quality of life; this has led to an increase in concern about the respiratory problems faced by ALS patients and a dramatic increase of relevant knowledge [7]. 

Several studies have shown that treatment of ALS patients with noninvasive positive pressure ventilation (NIPPV) for respiratory insufficiency appears to prolong life expectancy and improves the quality of life; this is possibly attributable to the slower rate of decline of pulmonary function in these patients [8]. These recent studies have demonstrated reduced mortality in ALS patients with respiratory complications and prolonged the average survival period through an increase in the use of respiratory assisting devices for managing respiratory problems. However, there is no effective treatment for respiratory dysfunction in ALS patients so far.

Acupuncture is one of the oldest medical interventions in East Asian countries. It has been shown in a recent study to be a safe and potentially effective intervention in patients with dyspnea, which is a major symptom of chronic obstructive pulmonary disease (COPD). Acupuncture has also been shown to be effective for symptoms in an animal model of ALS [9, 10]. Korean Sa-am acupuncture methods with lung tonification effects were chosen for this study after a review of the Korean traditional literature. K-ALSFRS-R scores, which are the main assessment tools of ALS, were used to analyze the relationship between the status of ALS patients and respiratory physiology parameters. The aim of this study was to report changes in EtCO_2_, SpO_2_, RR, and pulse rate, after Sa-am acupuncture treatment on ALS patients.

## 2. Methods

### 2.1. Subjects

This study was conducted at the Wonkwang University ALS clinic from January through July, 2012. Eighteen eligible ALS patients were selected out of all ALS patients admitted during that period. This study was approved by the institutional review board (IRB), and written informed consent was obtained from all participants.

 The inclusion criteria were as follows: patients who (1) satisfied El Escorial criteria and were diagnosed with ALS by EMG, (2) signed a consent, (3) cooperated with this study, (4) had not exercised within the previous 24 h, (5) had not smoked, drank alcohol, coffee, or green tea within the previous 8 h, (6) had eaten at least 1 h prior to testing, and (7) were not menstruating.

The exclusion criteria were as follows: patients who (1) needed intensive care for respiratory insufficiency, (2) were not able to give basic information owing to severe bulbar palsy, (3) had heart disease of ischemic or other etiology, (4) had endocrine disorders such as thyroid disease, (5) had renal diseases such as chronic renal failure, (6) had fever, (7) had a seizure disorder, (8) had mental illness, (9) were addicted to drugs such as alcohol, nicotine, or caffeine, and (10) were considered not eligible for this study at the discretion of the researcher. 

### 2.2. Measurements

#### 2.2.1. Measuring Devices: Capnography & Pulse Oximetry

Capnography & Pulse Oximetry (Nonin Medical, Japan) was chosen because it is easy to handle and is a useful measuring tool for observing changes in a patient's respiratory condition [7]. 

This study measured end-tidal carbon dioxide (EtCO_2_), peripheral oxygen saturation (SpO_2_), respiratory rate (RR), and pulse rate.

#### 2.2.2. K-ALSFRS-R

The Amyotrophic Lateral Sclerosis Functional Rating Scale (ALSFRS) is a validated, questionnaire-based scale that measures physical functional status in terms of the ability to carry out activities of daily living in patients with ALS. It has been used in clinical trials, as well as in clinical practice, because of its ease of use and its correlation both with objective measures of disease status and levels of disability [11]. The score reflects the deterioration of function in the natural course of ALS, but may have a lower sensitivity in advanced disease stages [12]. The scale was developed primarily to assess outcomes in pharmaceutical clinical trials and does not rely upon physical examinations or instruments [11, 13]. An initial imbalance within the scale that minimized the importance of respiratory function was rectified by a revision (ALS Functioning Rating Scale, revised [ALSFRS-R]) to incorporate respiratory symptoms and assess the need for ventilation [14]. The K-ALSFRS-R was made to reflect its domestic applicability; a preliminary experiment reported that it had high reliability and validity [15]. It consists of 4 sections (verbal, detailed motor, gross motor, and respiration function) and 12 subsections; the maximum possible score is 48 points. The greater the decrease in muscle function, the lower the score; in other words, higher scores reflect better patient status.

### 2.3. Procedures

**Figure 1:**
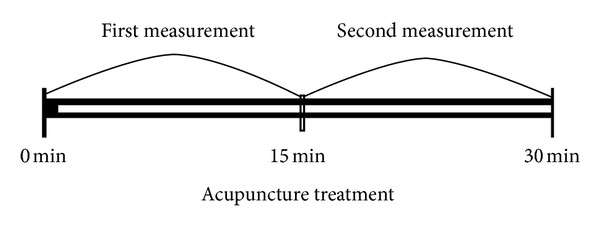
A pair of measurements in the trial. Respiratory parameters (EtCO_2_, SpO_2_, RR, and pulse rate) were measured using capnography and oximetry.

Experimental procedure was as follows: (1) subjects were advised to rest and not to undertake strenuous exercise before measurement, (2) respiratory parameters (EtCO_2_, SpO_2_, RR, and pulse rate) were measured using Capnography and Oximetry for 15 min, (3) Sa-am acupuncture was conducted at specific acupoints using 0.25 × 40 mm, sterile, disposable acupuncture needles made of stainless steel (DongBang Acupuncture Inc., Korea). The depth of insertion at each point was predetermined to be within the normal range of 8–20 mm, depending on the location of the point. The SP3 and LU9 acupoint needles were electrically stimulated at 100 Hz with the clinician adjusting the intensity so that the patients felt an uncomfortable sensation that was not painful; (4) needles were kept in place for 15 min while measuring EtCO_2_, SpO_2_, RR, and pulse rates simultaneously. The above process was considered as a pair of observations. Each patient received acupuncture treatment twice a day for 5 days (Figure 1).

#### 2.3.1. Selection of Acupuncture Points

**Figure 2:**
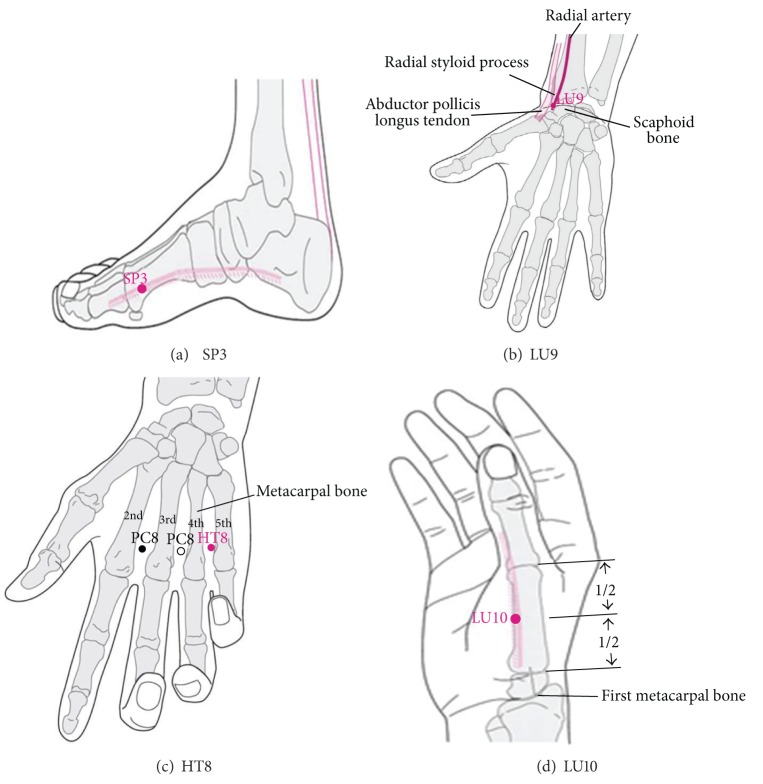
Locations of selected acupuncture points.

Acupuncture points SP3, LU9, HT8, and LU10 (Figure 2) were selected on both sides of the body according to Sa-am Five-Element Acupuncture method found in the traditional Korean medical literature. These sets of acupuncture points are used for the tonification of lung functions. Certified practitioners with at least 6 years of experience in traditional Korean medicine, and an additional 3 years of clinical experience, performed the acupuncture treatment according to the WHO Standard Acupuncture Point Locations for the Western Pacific Region. 

According to the Korean Sa-am acupuncture literature, SP3 (Figure 2(a)) and LU9 (Figure 2(b)) are used for the tonification of lung-*qi*, while HT8 (Figure 2(c)) and LU10 (Figure 2(d)) are used to clear lung fire. The selection of acupuncture points was based on the WHO standard acupuncture point guidelines [16]. 

### 2.4. Statistics

Statistical analysis using SPSS version 20.0 for Windows was used to compare changes in EtCO_2_, SpO_2_, RR, and pulse rate. The relationship between K-ALSFRS-R on one side and EtCO_2_, SpO_2_, RR, and pulse rate on the other was analyzed with Pearson's correlation analysis. To compare the difference between parameters of respiratory function before and after acupuncture stimulation, a paired *t*-test was conducted using the mean value over a period of 15 min. 

A significance level of *P* < 0.05 was used throughout.

## 3. Results

### 3.1. Demographic Characteristics

The sex, age, K-ALSFRS-R score, and site of onset of the included patients are given in Table 1. 

### 3.2. Correlation Analysis with K-ALSFRS-R Score

#### 3.2.1. The Analysis of the Values of the Differences of EtCO_**2**_, SpO_**2**_, RR, and Pulse Rate before and after Acupuncture Stimulation

**Figure 3:**
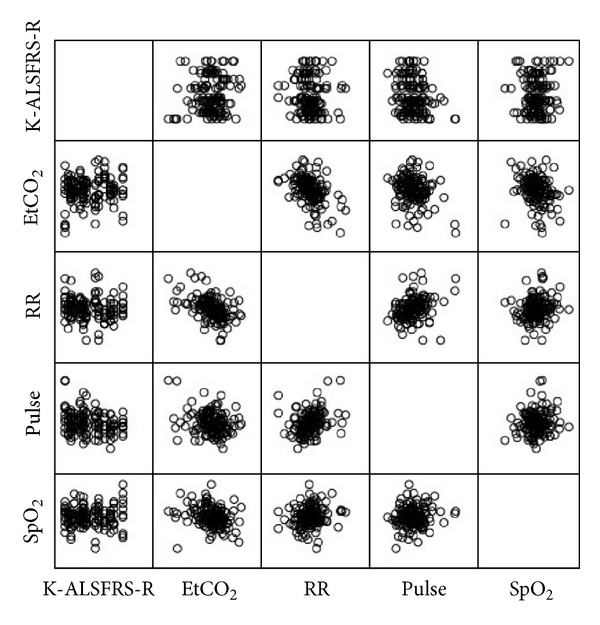
The correlation between K-ALSFRS-R score and the difference in EtCO_2_, SpO_2_, RR, and pulse before and after acupuncture stimulation.

Pearson's correlation analysis was used to analyze the relationship between K-ALSFRS-R score and differences in EtCO_2_, SpO_2_, RR, and pulse rate before and after acupuncture stimulation (Table 2, Figure 3). The results showed that there was a negative correlation between the K-ALSFRS-R score and the magnitude of increase in pulse rate after acupuncture stimulation (*r* = −0.236, *P* < 0.01). 

#### 3.2.2. Analysis of Respiratory Parameters after Acupuncture Stimulation

**Figure 4:**
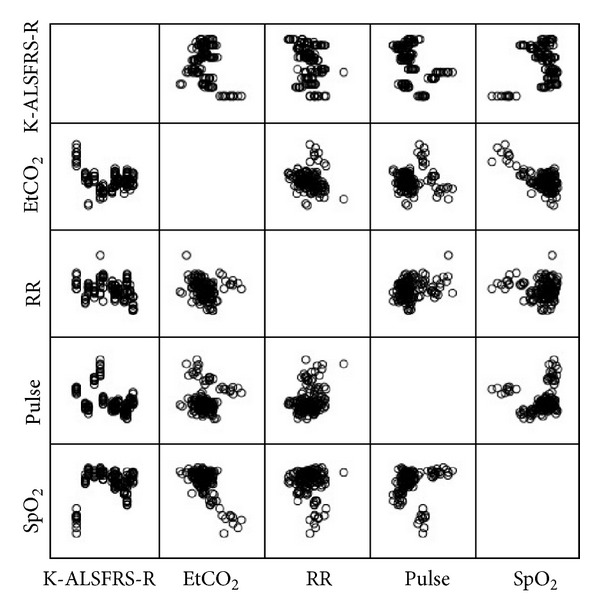
The correlation between K-ALSFRS-R score and change in EtCO_2_, SpO_2_, RR, and pulse rate after acupuncture stimulation.

Pearson's correlation analysis was used to show the relationship between K-ALSFRS-R score on one hand and EtCO_2_, SpO_2_, RR, and pulse rate on the other. (Table 3, Figure 4). There was a negative correlation between K-ALSFRS-R score and each of EtCO_2_ (*r* = −0.276, *P* < 0.01), RR (*r* = −0.254, *P* < 0.01), and pulse rate (*r* = −0.420, *P* < 0.001); that is, the greater the K-ALSFRS-R score, the greater the decrease in EtCO_2_, RR, and pulse rate after acupuncture stimulation. However, there was a positive correlation between the K-ALSFRS-R score and SpO_2_ (*r* = 0.173, *P* < 0.05), so that the greater the K-ALSFRS-R score, the greater were the increase in SpO_2_ after acupuncture stimulation. 

### 3.3. Changes in Respiratory Parameters before and after Sa-Am Acupuncture

**Figure 5:**
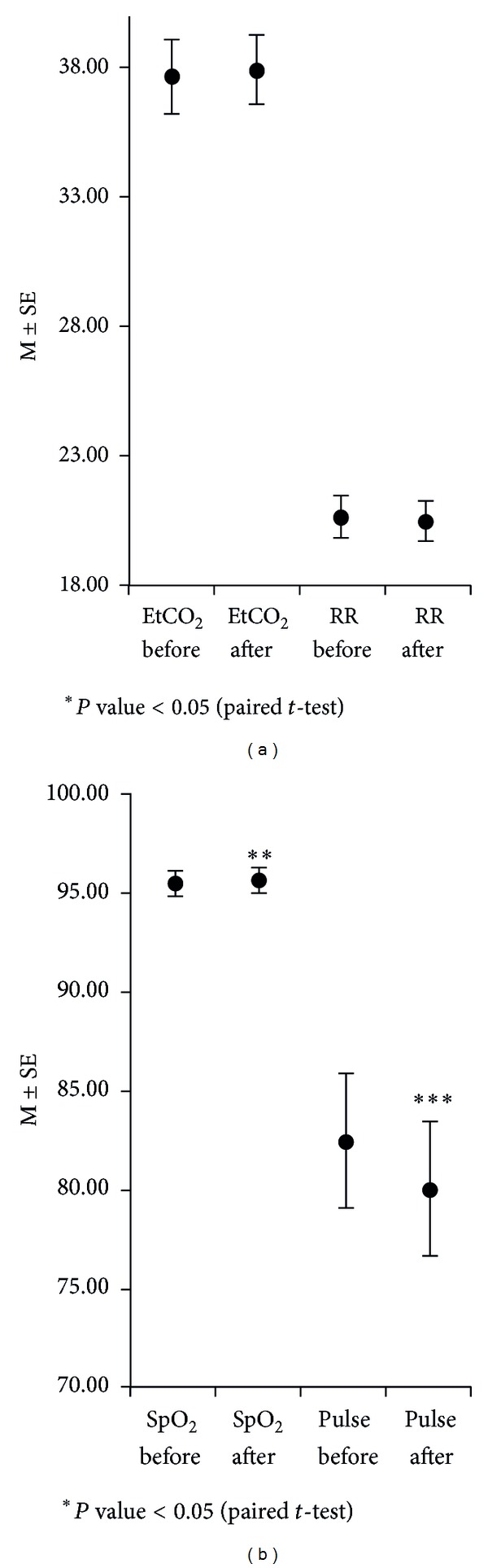
The changes in EtCO_2_, SpO_2_, RR, and pulse before and after Sa-am acupuncture.

Results obtained by performing a paired *t*-test showed no significant change in EtCO_2_ (*P* = 0.702 > 0.05) and respiratory rate (*P* = 0.180 > 0.05) after acupuncture stimulation. However, there was a significant difference between SpO_2_ before and after acupuncture stimulation, with an increase from 95.42% to 95.58% (*P* = 0.002 < 0.05). There was a significant change in pulse rate after acupuncture stimulation, with a decrease from 82.49 bpm to 80.08 bpm (*P* < 0.001) (Table 4, Figure 5). 

## 4. Discussion

ALS is a fatal and progressive neurodegenerative disease, leading to muscle weakness, paralysis, and death by respiratory failure. Although respiratory failure is generally the cause of death in ALS, little is known about the treatment and control of respiratory problems. Therefore, this pilot study was conducted to study the effect of acupuncture treatment on parameters of respiratory function with the help of capnography and oximetry to set up guidelines for preventing and managing respiratory problems in ALS. 

ALS belongs to the category of Wei symptoms in traditional East Asian medicine. The earliest published literature about Wei symptoms is “Plain Question” [17]. It reports several causes of Wei symptoms and suggests a treatment, which is focused on making the digestive system healthy [18]. Therefore, ALS patients have received acupuncture treatment specifically at the spleen meridian (SP) and stomach meridian (ST) in almost all case studies [19, 20]. 

Sa-am acupuncture, an original and traditional Korean acupuncture method, elicits a strong pain response when applied on the upper and lower extremities. Sa-am acupuncture is widely used in Korean clinical practice because it is considered safe and effective [21]; unfortunately, there has been little research on its use in ALS. 

According to this theory, the 4 causes of imbalances in meridian energies are deficiency, excess, cold, and heat. Since there are 12 meridians with 4 possible imbalances, there are a total of 48 preestablished acupuncture prescriptions, each with 5 transport points to use to restore these imbalances. The 5 transport points are important acupoints of meridian that control the 5-phase *qi* of viscera and bowels.

The acupoints of SP3, LU9, HT8, and LU10, which are referred to as lung tonification prescriptions, were selected as intensive acupuncture treatment in this study because respiratory muscle weakness and respiratory symptoms such as sputum production and shallow respiration are thought to be related to lung dysfunction.

Several studies have been conducted on acupuncture treatment in ALS. Byun et al. reported that shortness of breath improved by more than 50% after acupuncture treatment at HT8 and LU10 points on ALS patients [22]. Jiang et al. [10] reported that electroacupuncture could be an effective anti-inflammatory treatment for the respiratory impairment that occurs in animal models of ALS. However, almost all of these studies were conducted with small sample sizes, and the patients received not only acupuncture but also various traditional treatments. Moreover, some of these studies were performed on animal models of ALS and not on human patients. It is significant that the present study was conducted to observe the effect of acupuncture, with more patients recruited than ever before. 

It is known that EtCO_2_ exceeds 42 mm Hg when severe bronchial obstruction exists [23]. Therefore, the control of EtCO_2_ was considered an appropriate marker of an improvement of respiratory function and the alleviation of respiratory symptoms. Hypoxemia is defined as an SpO_2_ equal to or less than 93%, lasting for 15 s or longer; severe hypoxemia kills cells and suppresses mental activity, resulting in a comatose state and a reduction in the ability of muscles to perform work [24]. Dyspnea and hyperpnea are the most important symptoms of hypoxemia. 

A pulse rate of 60–100 beats/min and respiratory rate of 16–20 breaths/min are considered to be within the normal range in adults [25]. Acupuncture is able to modulate various autonomic responses [26]. A number of studies have shown impaired cardiac autonomic control in patients with ALS together with parasympathetic dysfunction and sympathetic predominance [27]. Disturbances in autonomic cardiac control in respirator-dependent patients with ALS may significantly influence survival and may lead to hypertensive crisis, circulatory collapse, and sudden death [28]. Observing and regulating EtCO_2_, SpO_2_, respiratory rate, and pulse rate can, therefore, be an important process of care for ALS patients. 

Analysis of K-ALSFRS-R score and parameters of respiratory function measured before and after acupuncture treatment showed that patients with high scores in K-ALSFRS-R had a greater difference in pulse rate before and after acupuncture stimulation. Patients who recorded high scores in K-ALSFRS-R showed a decrease in the values of EtCO_2_, RR, and pulse rate and an increase in the values of SpO_2_ after acupuncture stimulation. Overall results showed a high correlation with K-ALSFRS-R score and the therapeutic result of Sa-am acupuncture. There is rapid nerve degeneration as ALS progresses, therefore; acupuncture stimulation did not affect patients who were affected more severely. It is possible that Sa-am acupuncture would be more effective in the early stages of ALS.

There was a significant change in pulse and SpO_2_ after lung tonification by Sa-am acupuncture. Unfortunately, EtCO_2_ and RR showed no statistically significant changes despite a decrease at the time of measurement. Throughout this study, the results showed Sa-am lung tonification to be more effective in controlling inspiration rather than expiration. All patients who received Sa-am lung tonification showed a decrease in pulse rate; this suggests that Sa-am acupuncture treatment may play an important role in stabilizing sympathetic nerves and regulating the autonomic nervous system. However, acupuncture treatment achieved only a slight improvement in respiratory parameters; even then, it may help ALS patients to maintain respiratory function and retard the progression of respiratory muscle weakness. 

There were no side effects during the study; nevertheless, the study had its limitations. As a pilot study, there was no control group. ALS is a progressive and incurable disease, so it was not possible to distinguish between the treatment group and the nontreatment group for ethical reasons. It was also impossible to measure the effect of acupuncture treatment over long periods because these patients experience difficulty in breathing in the supine position. 

Research on the role of acupuncture in alleviating respiratory symptoms in ALS patients provides basic data for preventing respiratory complications, which generally lead to death in ALS patients. 

Ongoing research with the development and validation of new acupuncture treatment should continue in order to extend the life of ALS patients and improve their quality of life. 

## 5. Conclusion

Sa-am acupuncture led to a statistically significant difference in pulse rate and SpO_2_ after acupuncture stimulation. Patients in the earlier stages of the disease with high K-ALSFRS-R scores responded better to acupuncture treatment than did patients with lower K-ALSFRS-R scores.

This study needs to be taken further with a larger sample size to obtain more valuable and meaningful data.

## Figures and Tables

**Table 1:** Baseline characteristics of amyotrophic lateral sclerosis (ALS) patients in the study.

ALS patients	*N* = 18
Sex (male : female)	14 : 4
Age (years)	56.06 (±7.53)^a^
Age at onset	52.39 (±9.29)^a^
K-ALSFRS-R score (maximum: 48)	33.24 (±5.19)^a^
Respiration subscores in K-ALSFRS-R (total: 12)	9.71 (±3.24)^a^
Site of onset	
Bulbar	3
Upper limb	5
Lower limb	10

a: mean ± standard deviation.

K-ALSFRS-R: Korean-ALS Functional Rating Scale-Revised.

**Table 2:** The correlation between K-ALSFRS-R score and the difference of EtCO_2_,  SpO_2_,  RR, and pulse rate before and after acupuncture stimulation.

	EtCO_2_	SpO_2_	RR	Pulse
K-ALSFRS-R	0.104	0.046	−0.077	−0.236**

Analyzed by Pearson's correlation analysis (*P < 0.05, **P < 0.01).

EtCO_2_: end-tidal carbon dioxide, SpO_2_:  saturation of partial pressure arterial oxygen, and RR: respiratory rate.

**Table 3:** The correlation between K-ALSFRS-R score and the change in EtCO_2_, SpO_2_, RR, and pulse rate after acupuncture stimulation.

	EtCO_2_	SpO_2_	RR	Pulse
K-ALSFRS-R	−0.276**	0.173*	−0.254**	−0.420***

Analyzed by Pearson's correlation analysis (*P < 0.05, **P < 0.01, ***P < 0.001).

**Table 4:** The changes in  EtCO_2_,  SpO_2_,  RR,  and pulse rate before  and after Sa-am acupuncture.

	Before		After		*t*	*P*
	M	SD	M	SD		
EtCO_2_	37.70	5.47	37.94	5.35	−0.383	0.702
SpO_2_	95.42	2.48	95.58	2.57	−3.097	0.002**
RR	20.64	3.31	20.47	3.02	1.359	0.180
Pulse rate	82.49	13.33	80.08	13.15	9.992	0.000***

Analyzed by paired *t*-test (*P < 0.05, **P < 0.01, ***P < 0.001).
